# Pediatric Integrative Medicine in Academia: Stanford Children’s Experience

**DOI:** 10.3390/children5120168

**Published:** 2018-12-12

**Authors:** Gautam Ramesh, Dana Gerstbacher, Jenna Arruda, Brenda Golianu, John Mark, Ann Ming Yeh

**Affiliations:** 1 School of Medicine, University of California, San Diego, La Jolla, CA 92093, USA; gramesh@ucsd.edu; 2 Division of Rheumatology, Department of Pediatrics, School of Medicine, Stanford University, Palo Alto, CA 94304, USA; gerst1@stanford.edu; 3 Department of Pediatrics, School of Medicine, Stanford University, Palo Alto, CA 94304, USA; jarruda@stanford.edu; 4 Division of Pediatric Anesthesia and Pain Medicine, Department of Anesthesiology, Perioperative and Pain Medicine, School of Medicine, Stanford University, Palo Alto, CA 94304, USA; bgolianu@stanford.edu; 5 Division of Pulmonary Medicine, Department of Pediatrics, School of Medicine, Stanford University, Palo Alto, CA 94304, USA; jmark@stanford.edu; 6 Division of Gastroenterology, Hepatology and Nutrition, Department of Pediatrics, School of Medicine, Stanford University, Palo Alto, CA 94304, USA

**Keywords:** pediatric, integrative medicine, academic medicine

## Abstract

Pediatric integrative medicine is an emerging field which, to date, has not been described in detail in academic medical centers in the United States. Early research of pediatric integrative medicine modalities shows promise for the treatment of common pediatric conditions such as irritable bowel syndrome, acute and chronic pain, headache, and allergy, among others. In light of the growing prevalence of pediatric illnesses and patient complexity, it is crucial to emphasize the patient’s overall well-being. As academic centers around the world start to develop pediatric integrative medicine programs, the aim of this manuscript is to briefly highlight evidence of effective integrative treatments in pediatric subspecialties, to describe the establishment of our integrative medicine program, to summarize its early efforts, and to discuss potential barriers and keys to success.

## 1. Background

Integrative medicine (IM) is a patient-centric, evidence-based, therapeutic paradigm that coordinates the integration of all pertinent conventional and complementary approaches to achieve patient health. Integrative medicine incorporates all appropriate therapies, emphasizes the patient–provider relationship, and utilizes lifestyle changes to holistically optimize health and healing [1]. It addresses the biologic, psychosocial, spiritual, and environmental aspects of patient wellbeing. Integrative modalities range from mind–body interventions to nutrition. While current biomedical practices have made great strides in the treatment of many conditions, the addition of IM principles and practices can help restore the patient to a state of personal wellbeing and optimal function [2,3]. 

The National Center for Complementary and Integrative Health clarifies definitions frequently used with integrative medicine: complementary medicine refers to using non-allopathic medicine in conjunction with conventional allopathic medical treatment, and alternative medicine refers to utilizing a non-allopathic therapy instead of a conventional allopathic treatment. These two approaches are commonly referred to as complementary and alternative medicine (CAM) [4]. Throughout this article, the term “integrative medicine” (IM) or “IM practices” will be employed to refer to the concurrent use of CAM therapies in conjunction with traditional allopathic practice, in order to highlight this emerging practice paradigm in pediatric practice, except where the articles quoted have specifically employed the terminology of the terms “complementary” or “alternative” medicine.

The National Health Interview Survey results from 2012 show that 33% of adults and 12% of children used complementary health approaches [5,6]. Children with chronic illnesses are significantly more likely to use IM therapies than healthy children [7,8]. Integrative medicine is used by more than 50% of pediatric patients with chronic disease [2].

In the pediatric population, IM practices have enormous potential to reduce healthcare costs and ensure a healthier future for children by emphasizing prevention and promoting wellness [9]. The literature over the last two decades has confirmed the clinical efficacy of certain IM therapies and has demonstrated improved patient outcomes (e.g., improved quality of life, decreased cardiovascular risk factors), symptom relief, and patient satisfaction [10,11]. Large systematic reviews indicate potential cost-effectiveness and even cost savings for IM modalities in adult populations, though higher quality studies are needed [10]. Similar studies have not yet been published for pediatric patients but are currently underway [12].

By promoting healthy lifestyle practices early in the child’s life, pediatric IM sets a high standard for a lifetime of disease prevention behaviors. In many instances these therapies are simple and inexpensive to implement (e.g., improved sleep hygiene, exercise) and may provide long-lasting wellbeing. Exercise and lifestyle changes can treat depression, coronary heart disease, and type II diabetes [13,14,15,16,17,18]. Consuming a myriad of fruits, vegetables, spices, and extracts have shown preventative and treatment potential [19,20,21,22,23,24,25,26,27]. Mindfulness meditation has undergone extensive study utilizing functional magnetic resonance imaging (fMRI), and is emerging as an effective therapy for pain, anxiety, and depression [28,29,30,31,32,33]. 

The majority of integrative medicine users, however, do not report usage to their primary care physician; only 34–50% disclose use to their provider [34,35]. This number is likely even lower in certain ethnic and socioeconomic groups [1]. Parents and patients may feel intimidated by clinicians’ perceived negative attitudes towards alternative therapies, even though three in four pediatricians believe that parents would disclose this information [35]. Improved communication regarding IM therapies would allow for physicians to (1) understand patients’ and families’ priorities and values in health and illness, and (2) help avoid treatment interactions between CAM and conventional therapies [36]. 

It is important for pediatricians to have knowledge and facility with integrative medicine in order to initiate conversation about CAM utilization [37]. Helpful to the cause, guidelines for advising patients seeking CAM have been published [38,39]. Although clinicians have an ethical obligation to discuss treatment alternatives [40], the overwhelming majority of pediatric providers do not feel comfortable discussing CAM with their patients. Both Kemper and O’Connor [41] and Sawni and Thomas [42] found that less than 37% of pediatricians inquire regarding CAM use while taking a routine medical history; any dialogue regarding CAM is typically initiated by the family. Pediatricians have a positive attitude towards CAM, but desire further education to adequately address patient concerns [2,41]. In a national study of 648 pediatricians, less than one in five reported any formal training in CAM, and 84% of respondents desired a continuing medical education curriculum, exhibiting self-awareness of their low familiarity with the topic [3,41,42,43]. 

To meet the growing needs of patients and practitioners alike, representatives from eight institutions (including Stanford) met in Kalamazoo, Michigan in July 1999. The outcome of this conference was the creation of the Academic Consortium for Integrative Medicine and Health. The vision of this group was to support leaders in integrative medicine in their advocacy, expand familiarity with the field, and develop a scientific knowledge base and research opportunities. Most importantly, it aimed to establish a community of providers in this rapidly growing field of medicine. A survey in 2007 indicated that 16 of 143 North American medical schools had dedicated academic pediatric IM programs [44]. The growth of dedicated academic departments and training programs in IM highlight institutional interest and support for IM.

## 2. Pediatric Integrative Subspecialties in Academia

Academic medical centers are often the hub where education, research, and clinical care meet innovation to improve medical care. The field of pediatric integrative medicine thrives in this setting, which allows for collaboration across disciplines and the opportunity to treat medically complex patients across all socioeconomic and cultural backgrounds. Further, academic centers are conducive to the inception of novel scholarship and training initiatives.

The 2005 Institute of Medicine report recommended CAM education for all training levels in health professions schools [45]. From an educational standpoint, training in pediatric IM was spearheaded by the University of Arizona Center for Integrative Medicine. The 100-h online Pediatric Integrative Medicine in Residency (PIMR) program was designed to address the lack of IM training available to pediatric trainees [46]. The PIMR program teaches residents habits for their own personal wellness alongside an introduction to evidence-based IM principles. This program was adopted by five pediatric residency programs in 2012 as a pilot program. Inclusion of a curriculum on physician wellness in the PIMR program was intentional and necessary. Physician burnout and fatigue is associated with major medical errors and decreased productivity. Furthermore, it has been shown that healthy physicians are more likely to advise patients to follow healthy lifestyle habits and are more effective at motivating patients [47,48,49,50,51,52,53,54]. Embedding integrative medicine education in a pediatric academic setting has the additional advantage of increasing institution-wide exposure and comfort with IM modalities.

Pediatric IM is a much-needed subspecialty, emerging to meet the needs of today’s children. From a research standpoint, rigorous studies evaluating the safety, efficacy, and cost effectiveness of IM approaches in pediatrics are needed to justify usage [3]. Research into the efficacy of a pediatric integrative care model is often conducted at academic medical centers where patient diversity and research support are available. Due, in part, to increased research, IM approaches are being integrated into the fields of pediatric gastroenterology, pain, neurology, oncology, and pulmonology, among others. Frequently, the multidisciplinary approach afforded by IM can effectively treat symptoms, decrease polypharmacy, and enhance overall wellness in pediatric illness.

### 2.1. Gastroenterology

Pediatric gastroenterology (GI) patients have a high frequency of CAM usage; studies estimate utilization to be between 36% and 72% [55,56]. Herbal medicines, dietary supplements, and special diets are the most prevalent therapies utilized [57]. The strongest predictor of CAM use was prior or feared side effects of conventional medicine [55]. 

Integrative approaches for inflammatory bowel disease (IBD) include dietary assessment to identify food intolerances, dietary and herbal supplements, and mind–body therapies (relaxation techniques, meditation, and aromatherapy) [58]. Some studies have suggested a modest effect of probiotics, curcumin, and acupuncture in promoting remission in patients with IBD [59,60,61,62]. Mind–body interventions also have a limited body of evidence showing benefit in adolescents with IBD. A 2010 study of 67 adolescents with IBD described frequent utilization of prayer (62%), relaxation (40%), and imagery (21%) for disease management [63,64]. 

In patients with irritable bowel syndrome (IBS), there exists increasing evidence for the bidirectional connection between the mind, body, and gut. Gut-directed hypnotherapy (both in-person and home-based exercises) is also showing promise for pediatric IBS symptom relief [65,66,67]. Small studies on enteric coated peppermint oil have also demonstrated improvement of pediatric IBS symptoms [68].

### 2.2. Pain/Perioperative

Complementary and alternative medicine therapies for pediatric pain management are becoming increasingly accessible. A 2005 survey of 43 pediatric anesthesia fellowship programs approved by the Accreditation Council for Graduate Medical Education (ACGME) showed that nearly 90% of institutions had CAM therapies available for pediatric pain management. Common available modalities included biofeedback (65%), guided imagery (49%), and hypnosis (44%) [69].

Children and adolescents experience both acute and chronic pain, and each requires a different clinical approach. Nearly 40% of children with chronic pain experience persistent or recurrent pain at least once weekly, with common diagnoses being headaches, abdominal pain, and musculoskeletal pain [70]. Chronic pain can negatively impact a patient’s school functioning, sleep, and parent burden (e.g., financial obligations, missed work) [71]. Treating chronic pain requires a multidisciplinary approach. The complex interplay between the patient’s effective coping skills, parental reinforcement of pain behavior, and patient’s mood can all alter and affect patient functioning [71]. This patient demographic is, reassuringly, open to an integrative approach to their care. According to a study of 110 pediatric patients with chronic pain (headache, abdominal pain, and musculoskeletal pain) presenting at an academic integrative clinic, 100% of families were interested in additional counseling regarding diet, exercise, sleep, or stress management [70]. Further, evidence is emerging for non-pharmacologic treatments for pain. For instance, physical therapy (stretching, trigger point physiotherapy, massage) can relieve headaches, musculoskeletal pain, and chronic regional pain syndrome (CRPS) [72,73,74]. Cotton et al. found that after massage therapy, mean pain scores decreased in over 500 hospitalized children with chronic pain and anxiety [75]. Acupuncture and mindful meditation have been shown to be an effective treatment for chronic pain and can decrease opioid usage, but more robust randomized control trials (RCTs) are needed [29,76]. 

Acute pain in children can be experienced in acute illness, during procedures (including venipuncture or immunization), or post-operatively. Acute pain can also evolve into a chronic pain condition, requiring flexibility in diagnosis and management. Integrative modalities have been successfully used in the treatment of acute pain. For instance, acupuncture, clinical hypnosis, and breathing techniques have all been shown to improve pain scores in children with acute pain from illness or procedures [77,78,79]. Newer technologies, such as virtual reality devices, show significant promise in minimizing discomfort with vascular access and minor procedures [80,81]. 

### 2.3. Neurology/Neurodevelopmental Pediatrics

Pediatric patients with a wide variety of neurologic and neurodevelopmental conditions utilize CAM to alleviate symptoms and supplement conventional therapy. The use of CAM among pediatric neurology patients ranges from 24% to 78% [82,83]. In a survey of 327 pediatric neurology patients at the Mayo Clinic, over 40% used CAM, and melatonin for sleep disorders was the most commonly used therapy [84]. A 2014 survey of 206 pediatric neurology patients in a Canadian children’s hospital diagnosed with epilepsy, headache, and cerebral palsy illustrated high use of multivitamins, massage, and chiropractic therapies (89.9%, 47.1%, and 36.8%, respectively); most practices were reported as helpful [85]. Among children with autism spectrum disorder (ASD), special diets are the most common form of CAM utilized according to eight independent studies [57,86]. 

In pediatric headache patients, headache frequency and intensity has been shown to improve with magnesium and riboflavin (vitamin B2) [87,88]. Furthermore, screening and treating comorbid sleep disorders such as sleep apnea, bruxism, insomnia, and restless leg syndrome can indirectly improve headache [89]. Neurofeedback has documented potential in treatment of epilepsy and headache [90,91]. Meditation, breath work, and relaxation activities have reduced school absences in patients with headaches, migraines, and seizures [83]. 

### 2.4. Pediatric Oncology

Between 1977 and 2011, 31 studies on the use of IM practices in pediatric oncology patients listed spirituality/prayer, positive mental imagery, and natural health products (multivitamins, megavitamins and herbals) as common interventions [57]. Mind–body therapies are readily accessible and low risk, with several trials showing decreased side effects of cancer therapy, increased patient self-confidence, and improved coping skills [92]. The largest study of pediatric oncology patients (1063 patients) found that 71% utilized IM modalities and only 4% reported adverse events [93]. The most common reasons cited by patients for using IM were to aid in treating cancer, symptomatic relief, support of conventional treatment, and management of side effects [94,95].

Integrative modalities may also play a role in long-term cancer risk reduction. Patient lifestyle factors such as stress, cigarette smoking, poor diet, alcohol consumption, and a sedentary lifestyle are revealed and discussed with greater frequency in the IM approach. Research has suggested numerous cancers to be primarily nutrition-responsive and preventable by dietary [96,97] and lifestyle changes [98,99]. The mechanism responsible may be by aliment-induced reversible epigenetic modification or the protective effects conferred by a diet high in fiber and antioxidants [20,100,101]. Establishing healthy habits in children to focus on healthy nutrition, mind–body interventions for stress reduction, quality sleep, and generous physical activity may be paramount in decreasing overall risk of some cancers (colon, lung, breast, and prostate) in later life [102].

### 2.5. Asthma and Allergy

Pediatric asthma is another area where an integrative approach may provide benefit. Elimination diets and those emphasizing core facets of the Mediterranean diet (high in fruits, vegetables, and legumes) have been shown to protect children from asthma and allergies [103,104,105]. A systematic review and meta-analysis by Cramer et al. in 2014 highlighted yoga as a supplementary intervention for asthma patients to improve symptoms and quality of life [106]. Breathing exercises and breath retraining techniques such as Buteyko breathing, yoga/pranayama, and physiotherapy have decreased bronchodilator usage [107,108]. Mark describes a multitude of integrative therapies as effective for pediatric asthma, including: (1) mind–body therapies to reduce anxiety and stress, lowering immune response and sympathetic activity; (2) proper prenatal and childhood nutrition that is high in fruits and vegetables; and (3) exercise and yoga to improve regimen adherence and decrease anxiety [109,110].

### 2.6. Other Subspecialties

There is a role for an integrative approach within many subspecialties. We know, for instance, that diet and exercise management can halt or reverse cardiovascular disease, diabetes, and obesity [10,13,23,111,112,113]. Integrative cardiology targets high body mass index (BMI) and poor nutrition using mind–body therapies to influence caloric consumption, sedentary or stressful lifestyles, and depression-associated symptoms [114]. Cognitive behavioral therapy has been used as an adjuvant in pediatric obesity management [115]. Mindfulness-based cognitive therapy has shown preliminary efficacy in youths with anxiety disorder and mindfulness based stress reduction programs in urban youth have improved psychological functioning and decreased negative effects of stress [33,116]. Furthermore, a recent study has proposed mechanistic pathways of action of acupuncture in alleviating cardiovascular disease [117,118,119,120].

As the evidence base to support the use of integrative approaches in each pediatric subspecialty grows, the use of CAM in pediatrics will increase. In light of this, clinical practices and academic centers are starting to integrate CAM modalities. Existing models of care can provide a foundation for future growth and utilization of integrative approaches for children. 

## 3. Integrative Medicine at Stanford Children’s Health

This section presents the historical development of Pediatric Integrative Medicine at Stanford Children’s Health with the intent to afford inspiration and perspective for future programs in pediatric IM.

### 3.1. Program Background and History

Lucile Packard Children’s Hospital and Stanford University School of Medicine are located in Palo Alto, California and serve the broader region of the San Francisco Bay area. The inpatient children’s hospital houses 361 beds and outpatient clinics serve over 500,000 visits annually. Families travel from 50 states and 40 countries for care. Fifty-two percent of families have private insurance, while 45% have public health insurance.

The Pediatric Integrative Medicine (PIM) program was established at Stanford Children’s Health in 2011 with support from the pediatric department leadership. The program was developed in recognition of the growing interests of patients and their families in the use, benefits, and potential complications of complementary therapies. The program’s first activity was to survey all Stanford Children’s Health medical providers (including nurse practitioners, residents, fellows, and attending physicians) and measure their knowledge of and interest in IM. The survey revealed that a majority of practitioners (over 75%) wished to learn more about IM. Reasons for wanting further education included: (1) a desire to learn more about different CAM therapies to better advise their patients and families about safety and efficacy of supplements and mind–body approaches (which they were already using); and (2) a need to knowledgably introduce evidence-based pediatric IM practices into patient care. 

**Figure 1:**
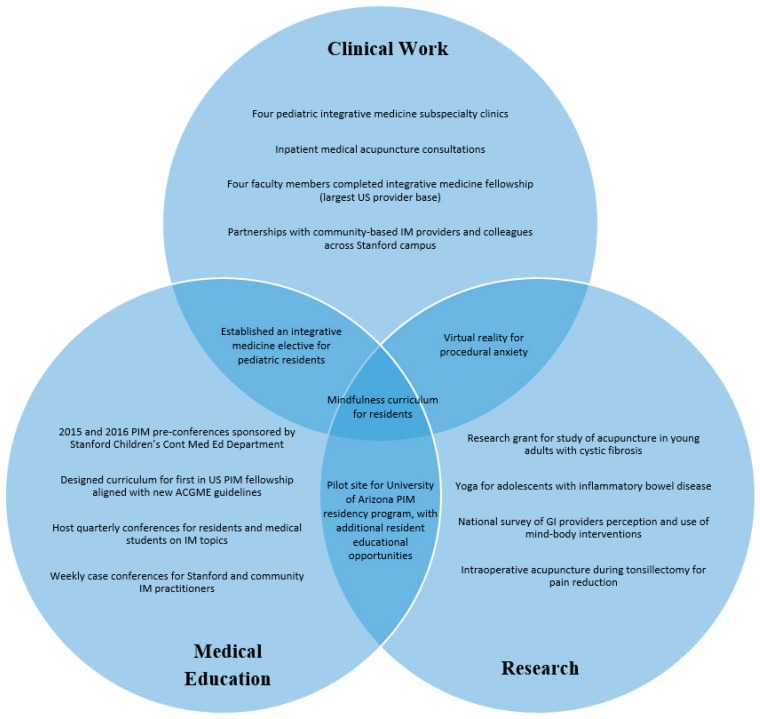
Program accomplishments. IM: integrative medicine; PIM: pediatric IM; GI: gastrointestinal; ACGME: Accreditation Council for Graduate Medical Education.

The PIM faculty prepared a business plan to ensure the new program’s sustainability and improve access for families seeking expertise in these therapies. The business plan included: proposed faculty, support staff allocation (0.5 days per week), and projected revenue from clinical services. Rather than creating a separate PIM clinic, the four faculty members centered the integrative clinics within four established specialty clinics: pain, gastroenterology (GI), pulmonary, and rheumatology. This model aimed to decrease overhead cost (space, staff, supplies) and make PIM clinics easily accessible. This model was fiscally sustainable as it allowed continued financial support of the faculty for conventional work in their respective subspecialty in addition to the subspecialty PIM clinics. The anecdotal response to this model by patients, families, and institutional leadership was positive. Patients and families expressed satisfaction (e.g., >80% provider satisfaction scores on yearly Press Ganey reports) and gratitude for finding pediatric subspecialists that were knowledgeable in both mainstream treatments and integrative modalities. As the program grew, institutional leadership and division chiefs continued to support the program, allowing the IM faculty to shift more time toward integrative clinics. In addition, as pediatric providers in the community learned of the PIM faculty’s expertise, they began referring families for consultation. Community providers were welcomed to educational sessions offered by the program leaders and began incorporating evidence-based IM practices into their own specialties. Figure 1 is a diagram of the program’s accomplishments.

### 3.2. Clinical Work

The four subspecialty integrative clinics (staffed by four IM-trained faculties in the pulmonary, rheumatology, pain medicine, and gastroenterology fields), continue to grow. Clinical volume has increased significantly since inception in 2012. In 2016, there were 160 visits for integrative medicine, and by 2017 that number had nearly doubled to over 300 annual visits. The greatest growth was noted in integrative pain and gastroenterology clinics. Referrals have come from community alternative care providers (e.g., Chinese medicine practitioners and naturopathic practitioners), community pediatricians, providers within Stanford Children’s Health, and self-referrals. Approximately 25% of encounters are new patient visits and the rest are follow-up visits. 

Prior to an initial visit with a PIM provider, insurance authorization is obtained. If the visit is with an acupuncture provider, patients interested in acupuncture are given current procedure terminology (CPT) codes and instructed to determine insurance benefits and out-of-pocket costs. Many insurances in California do cover acupuncture services for certain indications. Authorization is also required for pain psychology services through the IM pain clinic. An insurance authorization team is available to assist in this process. 

Each subspecialty clinic has a slightly different staffing structure. The integrative pain clinic includes a comprehensive initial pain evaluation for each new patient with the pain physician, nurse practitioner (NP), and pain psychologist. Depending on patient’s needs, subsequent follow-up visits are with the pain medical doctor (MD)/NP and either the Stanford pain psychologist or community psychologist. Pain clinic NPs are also medical acupuncture-trained and therefore can continue treatment plans started by the acupuncture-trained physician. New patient visits in IM GI, pulmonary, and rheumatology clinics are for 90 min with an IM physician and follow-up visits are for 45 min. After a through intake evaluation, a comprehensive treatment plan is developed. The modalities offered depend largely on the individual training of the providers. The range of integrative treatment modalities include: nutrition recommendations, mind–body interventions, acupuncture, botanical or supplement review, and recommendations. The integrative GI clinic and integrative pulmonary clinic both have access to a registered dietician and social worker directly within the clinic. Pediatric IM providers will often refer to Stanford colleagues in the fields of nutrition, occupational therapy, psychiatry, child and pain psychology, and physical therapy. Frequency of referral to these services depends somewhat on the subspecialty, but the highest utilized services include psychology and occupational therapy for biofeedback. Further, the IM faculty will refer to community providers outside of the institution in acupuncture, massage, physical therapy, biofeedback, clinical hypnosis, mindfulness, and yoga. Community referrals are especially common when a patient lives a far distance from the Stanford campus and needs a particular therapy frequently (for example, weekly acupuncture for a patient who lives three hours away).

In the inpatient setting, a few IM modalities are available to inpatients through existing clinical departments. For example, the inpatient pain service provides acupressure and acupuncture. The child life department provides mind–body therapies, including guided imagery and virtual reality. The child psychology service provides clinical hypnosis as one of their treatment interventions. At this point there is not a dedicated PIM inpatient service but rather a meshwork of services offered by individual departments. For the future, an inpatient IM consultation service is a planned area for program expansion. 

### 3.3. Research

Research for many integrative modalities is lacking, especially for pediatric patients. The Stanford faculties have produced a variety of publications including narrative reviews, book chapters, prospective pilot studies, retrospective studies of clinic outcomes, and position statements. Three faculty members are in the clinician educator track which does not afford protected research time. One faculty member is in the physician scientist track and has a dedicated 0.25 full time equivalent (FTE) of research time. Several generous foundation grants (Lawlor Foundation and Mayday Foundation) have allowed for support of the faculty and research assistants. Notable scholarly work includes faculty collaboration with PIM thought leaders to produce an Academy of Pediatrics (AAP) position statement on pediatric integrative medicine [3] and detailed results from the Pediatric Integrative Medicine in Residency (PIMR) pilot program [46,121]. A recent study found immersive virtual reality safe and effective for treating complex regional pain syndrome [80,122]. This has prompted further research on using virtual reality for other types of chronic, acute, and procedural pain. Intraoperative acupuncture for patients undergoing tonsillectomy and adenoidectomy was found to be feasible, decreased postoperative pain, and increased return of diet [77]. A pilot study on yoga for adolescents with inflammatory bowel disease found yoga to be widely acceptable, feasible, and safe [123]. For further details and a summary on research productivity and ongoing projects please see Table 1. 

Challenges to studying the PIM program as a whole is that program providers often recommend a comprehensive treatment plan instead of a single intervention. This mirrors the wide array of interventions offered at other programs around the country (particularly for pain clinics) [69,124]. Therefore, developing research techniques to study multimodal interventions is needed to adequately study the integrative medicine approach as a whole [125]. This may include examining cost effectiveness and resource utilization (emergency visits, urgent care visits, and hospitalizations) in patients seen in a PIM program versus standard care.

### 3.4. Medical Education

Stanford pediatric residents began participating in the University of Arizona’s Pediatric Integrative Medicine Residency (PIMR) pilot online training curriculum in 2014. Trainee participation in this curriculum was a pivotal step towards formalizing PIM medical education efforts at Stanford Children’s. In addition to providing a foundation in PIM knowledge, the curriculum enabled residents to improve their own lifestyle and wellness behaviors during the pilot [121]. To supplement the online curriculum offered by PIMR, the faculty developed a pediatric integrative medicine and wellness elective for pediatric residents who wanted in-depth exposure to integrative medicine in practice. The elective is currently offered as either a two-week or four-week in-person rotation. Residents taking the elective participate in the subspecialty PIM clinics (average four to five half-day clinic sessions per two-week rotation). Required readings and hands-on didactic sessions (average 4 h per two-week rotation) include lectures about plant-based nutrition and mind–body medicine. A field trip to a natural foods store provides the venue for a hands-on discussion of herbs and supplements. Residents are also asked to choose one integrative modality to experience for themselves to improve their own health. Meditation, massage, acupuncture, and yoga have been the most popular modalities that residents explore. Residents enrolled in the four-week elective are required to complete the 100-h PIMR online curriculum in addition to the above activities. The PIM elective at Stanford Children’s has become one of the most popular electives, with approximately 20% of the 76 categorical pediatric residents participating annually. 

At the fellow physician level, a new wellness curriculum has been developed by the Stanford Children’s faculty, including the PIM faculty. The Fellowship WellBeing Program (FWP) focuses on fatigue mitigation, self-care, resiliency, and stress mitigation for over 100 pediatric fellows. This seven-hour curriculum emphasizes the use of breathing, movement, mindfulness and nutrition to help physician trainees find and maintain wellness. 

In 2018, Stanford Children’s launched the first one-year clinical fellowship in pediatric integrative medicine to formally train pediatricians in integrative medicine. The PIM fellowship includes partnership with the University of Arizona Fellowship in Integrative Medicine, a 1000-h, two-year distance learning program with three, week-long, hands-on training sessions. The fellow has a dedicated general pediatrics IM clinic precepted by the PIM faculty (average two half-day clinics per week) and also works alongside faculty members in their respective subspecialty PIM clinics (average two half-day clinics per week). Curriculum intensives—one to three-week mini courses—were developed by the PIM and adjunct faculty members to cover the important PIM topics of nutrition, mind–body medicine, botanical medicine, and inpatient consultation. The fellow may also work with non-Stanford affiliated community integrative medicine pediatric providers on an elective basis for niche skill development and clinical exposure. The fellow is also required to teach pediatric residents formally in several conferences per year and informally when residents are on the PIM elective. The fellowship curriculum includes weekly didactic and case conferences in the style of “Professor’s Rounds” where a patient case is discussed with providers from various medical backgrounds and training expertise. The conferences are attended by community integrative medicine general pediatricians, PIM subspecialty providers (GI, pulmonary, pain, rheumatology), massage therapists, acupuncturists, psychologists, mind–body intervention providers, and nutritionists. After each case presentation, attendees offer their treatment recommendations (from their own unique perspectives), and these recommendations are provided to the patient at clinic follow-up. 

#### Sample Case Presentation (Identifying Details Changed for Patient Privacy)

A 15-year-old girl presented to the outpatient pediatric integrative medicine clinic with chief complaint of abdominal pain for three years which had been diagnosed as abdominal migraines. A comprehensive history was taken regarding her pain including an evaluation of life stressors, a detailed diet history, and her extensive medication list.

Pertinent findings included a past medical history of anxiety and release of a tethered cord at nine months of age. Her prescription medications included duloxetine, cariprazine, amitriptyline, topiramate, polyethylene glycol, and a combined estrogen–progesterone oral contraceptive pill daily. She took clonazepam, ondansetron, sumatriptan, cyproheptadine, and simethicone on an as-needed basis for symptoms related to abdominal migraine. She did not find any of these as needed medications particularly helpful in treating her abdominal migraine episodes. Supplements included melatonin nightly and peppermint oil by mouth as needed for abdominal pain. She was an only child of her mother and father. She was starting the 10th grade and achieved good grades. Her bedtime was 22:00 h nightly and she fell asleep easily. She reported occasional overnight awakenings and feeling tired on waking at 06:00 h daily. She described herself as a worrier but also as willful and ambitious. Her favorite color was teal, and her favorite season was winter. She preferred salty foods. Both mom and patient readily offered that the patient’s desire to please her teachers often led to high anxiety and tears if she felt like she was not working to her potential due to illness. Her self-reported personal strength was relating to other people, including classmates. She stated she was weak in mathematics. Her physical activity included biking to school or walking to the school bus stop. She was planning to join the school speech and debate team at the time of the visit. 

On physical exam her weight was in the 73rd percentile and her height was in the 38th percentile. Her body mass index was 22. She was talkative and engaged. Abdominal exam was significant for hyperesthesia with light touch of all abdominal quadrants with significant epigastric tenderness. There was no palpable stool burden. The remainder of her physical exam was normal. Prior negative workup for her abdominal pain included infectious stool studies, *Helicobacter pylori*, fecal calprotectin, complete blood count with differential, and a comprehensive metabolic panel including liver function testing, amylase, lipase, sedimentation rate, lipids, tissue transglutaminase immunoglobulin A, ceruloplasmin, and thyroid stimulation hormone. The results of these studies were normal.

The patient’s comprehensive treatment plan included mind–body, lifestyle, and diet recommendations. Mind–body therapies were discussed, and the patient elected to start attending a free yoga class at her primary medical center. She continued to see her outpatient psychiatrist for weekly psychotherapy including cognitive behavioral therapy. From a lifestyle perspective, she was recommended to increase physical activity and offered that she would start walking a few evenings per week with her mother. A therapy plan for acute abdominal discomfort was formulated, and, in addition to her current pharmaceutical regimen, included: acupressure massage, enteric coated peppermint oil, and aromatherapy. 

At her first follow-up the patient reported acupressure massage helpful, and she and the family asked for further instruction on in-home use of acupressure massage. At the third visit, approximately six weeks later, the patient emphatically reported she had aborted two abdominal migraines using acupressure beads with massage—something she had never achieved before. She started receiving biweekly acupuncture. She continued yoga once per week. She also found benefit from using a mind–body application on her mobile phone for daily meditation to augment anxiety treatment. She weaned off amitriptyline and topiramate without incident and reserved only ondansetron and clonazepam on an as needed basis for acute discomfort. In the first three months of treatment the patient aborted abdominal migraines twice and experienced only two breakthrough episodes. Additionally, she missed fewer days of school due to abdominal pain. She continues to follow-up in the integrative medicine clinic every two–three weeks for acupuncture treatments. 

## 4. Discussion 

### 4.1. Pediatric Integrative Medicine in Academia

Pediatric Integrative Medicine is an emerging subspecialty that provides the foundation for whole-patient and whole-child preventative care and lifestyle medicine [44]. Within children’s hospitals, patients are acutely sick, and in the outpatient setting, the incidence of children with complex and chronic medical problems has grown. According to a national survey performed by the Centers for Disease Control, in 2009, 15.1% of all children in the United States had special health care needs, up from 12.8% in 2001 [137]. These children often have multiple subspecialties involved in their care and their subsequent care coordination and communication between multiple consultants can be challenging. Specialists tend to focus on their organ system of interest, and the holistic approach to the care and healing of the child may be overlooked. Integrative medicine may help bring together all aspects of care since PIM is a blend of mainstream therapies with the other aspects of wellness. Further, our PIM faculty members are trained in both a pediatric subspecialty and integrative medicine. This affords a unique opportunity for specialized clinical care, education, and research in these integrative pediatric subspecialties. 

### 4.2. Drivers for Success

A primary reason for our program’s initial success was due to institutional support, strong leadership, and a financial model of embedding the integrative clinics within subspecialty divisions. Institutional leadership recognized that the PIM program is an attractive feature that is “uniquely Stanford” and serves the local San Francisco Bay Area community and beyond. 

In addition, recruiting and collaborating with the well-respected existing faculty within the institution to provide consultations (rather than hiring or contracting with external providers) established trust, garnered respect for the program, and continued collegiality amongst providers. This mirrors an important key to success among other established non-pediatric integrative medicine centers around the world [44,138]. Given that integrative medicine at times can include treatment modalities that may not be well known to mainstream medicine, the faculty group also emphasizes evidence-informed treatment modalities to the extent available and emphasizes patient safety. Lifestyle recommendations that have minimal side effects such as good nutrition, sleep hygiene, physical activity, and mind–body modalities are often cornerstones of each treatment plan. When research on treatment efficacy in a pediatric population may not be available, providers extrapolate data from adult populations and utilize shared-decision making with families, and use the safety-effectiveness rubric to discuss and document efficacy [139]. Every effort is made to utilize available resources to ensure the safety of a treatment recommendation, especially in regard to botanicals or supplements. Further, pharmacy consultations are sometimes required in patients who have significant polypharmacy. 

The medical education initiatives jumpstarted awareness of integrative medicine throughout the children’s hospital faculty and leadership. The resident PIMR pilot and subsequent PIM elective required residency leadership approval of the residency curriculum change. The elective also increased resident exposure and engagement with the integrative medicine faculty, which led to several resident-initiated scholarly works [123,132]. The novel conception of the pediatric integrative medicine fellowship has additionally increased clinical services and education efforts. The weekly didactic conference (available in person and by webinar) had the unanticipated benefit of bringing together PIM providers within Stanford and the larger community. Continuing education courses for the pediatric community also stimulated community building and aided in patient referrals. As students, residents, and other community providers increase their exposure to and knowledge of integrative modalities, these treatment options become part of the norm, and then truly “integrated” into conventional care. Therefore, medical education and teaching are important keys to success.

Finally, the educational and research programs would not exist without generous philanthropic support—both in the form of private donations and research grants. These funds aided to establish funding for the program’s research and have allowed the pediatric integrative medicine fellow to attend the Arizona Center for Integrative Medicine’s distance learning fellowship.

### 4.3. Challenges and Financial Considerations

The program has faced several challenges. From a financial standpoint, integrative medicine bills using time-based evaluation and management codes, behavioral health codes, and acupuncture CPT codes. Although we have not had significant challenges having physician billing codes covered, preventative services in the current fee-for-service model do not reimburse equally when more time is spent with the patient (compared to seeing several patients in the same time period). Currently, the PIM faculty members work in their respective pediatric subspecialties to offset some of the costs of the PIM clinic. A separate bill center was created to track financial progress and will add insight to this challenge. As newer models of care such as accountable care organizations (ACOs) are adopted in adult primary care fields [140], this will hopefully translate to novel prevention models for pediatrics. 

While children from underserved populations are still able to be seen by PIM providers at our institution, additional services such as acupuncture, biofeedback, psychology, and nutrition may not be as readily covered. Further, these patients may not be able to afford additional supplements and suggested dietary changes. This continues to pose a significant challenge for our underserved population and deserves significant and sustained advocacy and philanthropy.

While the newly developed PIM fellowship currently has institutional and philanthropic support, the sustainability of this training program is uncertain. Given that it is a completely new fellowship program, accreditation by the ACGME is likely years away. Currently, a two-year pilot by the hospital and Pediatrics Department is currently funding the fellow salary, program director effort, and coordinator time; philanthropy is funding the educational programming for the fellow.

Lastly, the concept of an integrative approach is, at times, a difficult philosophical mindset for patients and families. Behavior, lifestyle, and diet changes are frequently more challenging to implement than taking medications. These changes also take time. Referring providers and patients may be looking for a “quick fix”, which may not be feasible. While evidence on integrative modalities is emerging in pediatrics, colleagues often remain skeptical and have reservations on the value of integrative therapies. 

### 4.4. Future Directions

The PIM program at Stanford hopes to increase clinical services, education efforts, and research productivity. Clinically, a pilot inpatient integrative medicine consultation service is planned for Spring 2019. Discussions are underway to integrate mind–body and acupuncture treatment modalities to perioperative and postoperative treatment protocols. The program also needs to formally survey patients and families on patient satisfaction of its current programs. 

On a national level, the faculty aims to collaborate with national organizations such as the American Academy of Pediatrics and other pediatric subspecialty organizations to have a broader reach for educational efforts. Several faculties have participated in discussions about developing an integrative medicine core competency requirement for all pediatric residents. Additionally, our program leadership advocates for other academic centers to establish pediatric integrative medicine clinical training fellowships. 

Continued research is needed to establish evidenced-based safety and efficacy data for integrative therapies in pediatrics. Specific ongoing projects at our center include examining acupuncture for patients undergoing craniotomy, mind–body interventions for pediatric inflammatory bowel disease, and utilizing virtual reality for procedural pain and anxiety. Our program would be open to collaborating with other centers to develop the recently proposed multi-center PIM research network [139]. Continued growth in these areas will require ongoing institutional support for faculty time, resource allocation, and financial support. 

## 5. Conclusions

Evidence of safety and efficacy of pediatric integrative treatment modalities within pediatric subspecialties continues to grow. The establishment of a pediatric integrative medicine program within an academic setting is feasible. It requires sufficient institutional support, funding, and adequately trained physician faculty and staff. Important contributors to our program’s success include medical education to drive provider and trainee education, an emphasis on patient safety and evidence-based medicine, and an incredible team of enthusiastic physician leaders.

## Figures and Tables

**Table 1:** Summary of Published Scholarly Work on Integrative Medicine.

Category	Name of Study	Study Type	Findings	Funding
Pediatric integrative medicine	Pediatric Integrative Medicine [3]	Position Statement	Position Statement of Pediatric Integrative Medicine	N/A
Medical education	Pediatric Integrative Medicine in Residency (PIMR): Description of a New Online Educational Curriculum [46]	Retrospective review	Online curriculum targets integrative medicine (IM) knowledge gaps in pediatric residents	Funding received from foundation grants outside of Stanford
	Pediatric Integrative Medicine in Residency Program: Relationship between Lifestyle Behaviors and Burnout and Wellbeing Measures in First-Year Residents [121]	Retrospective review	Details burnout wellbeing in PIMR participants	Funding received from foundation grants outside of Stanford
Pediatric pulmonary	Complementary and Alternative Medicine in Pulmonology [126]	Literature review	Examines complementary and integrative medicine (CAM) use and effectiveness in children with pulmonary disorders	N/A
	Integrative Medicine in Asthma [127]	Literature review	Details integrative approach for children with asthma	N/A
	Nutrition in Pediatric Cystic Fibrosis [128]	Book chapter	Details evidence of nutritional therapies in children with cystic fibrosis	N/A
	Integrated Medicine and Asthma [129]	Book chapter	Details evidence of integrative approach to asthma	N/A
Pediatric gastroenterology	Yoga as adjunct therapy for adolescents with inflammatory bowel disease: A pilot clinical trial [127]	Prospective pilot	Yoga is acceptable, safe and feasible for adolescents with IBD	Tracie Lawlor Foundation, prAna
	Mind-Body Interventions for Pediatric Inflammatory Bowel Disease [130]	Literature review	Review of evidence on mind-body interventions for IBD	N/A
	Integrative Treatment of Reflux and Functional Dyspepsia in Children [130]	Case study and literature review	Describes integrative approach to children with reflux and functional dyspepsia	N/A
	Acupuncture and Integrative Medicine for Pediatric Gastroesophageal Reflux and Functional Dyspepsia [131]	Retrospective case series	Describes effect of acupuncture on children with GERD and functional dyspepsia	Stanford Medical Scholars Program
Pediatric neurology	Integrative Medicine in Child Neurology: what do providers think and what do they need to learn? [132]	National survey	Describes results of a national survey of IM modalities used in pediatric neurology	N/A
Pediatric pain	The Impact of Massage and Reading on Children’s Pain and Anxiety After Cardiovascular Surgery: A Pilot Study [133]	Randomized prospective trial	Massage was safe and feasible for children undergoing cardiac surgery. Massage decreased anxiety scores and lowered exposure to benzodiazepines.	N/A
	Immersive Virtual Reality for Pediatric Pain [80]	Literature review	Review of evidence on using virtual reality for acute, chronic, and procedural pain	Mayday Foundation
	Two Virtual Reality Pilot Studies for the Treatment of Pediatric CRPS [122]	Prospective pilots	Virtual Reality feasible and effective for treating pediatric CRPS	Mayday Foundation
	Non-Pharmacological Techniques for Pain Management in Neonates [134]	Literature review	Details evidence of non-pharmacologic techniques to treat neonatal pain	N/A
Acupuncture	Acupuncture as an Anesthetic Adjuvant for Pediatric Orthopedic Patients: A Pilot Study and Protocol Description [79]	Prospective pilot	Acupuncture was associated with low pain scores and levels of nausea in patients undergoing orthopedic surgery	N/A
	Does Acupuncture Reduce Stress Over Time? A Clinical Heart Rate Variability Study in Hypertensive Patients [135]	Retrospective case study	Acupuncture increased heart rate variability after acupuncture treatment in adults undergoing treatment for hypertension	N/A
	Does Noninvasive Electrical Stimulation of Acupuncture Points Reduce Heelstick Pain in Neonates [136]	Randomized prospective trial	Noninvasive electrical stimulation at acupuncture points was not effective to decrease heelstick pain in neonates	Mayday Foundation
	Intraoperative acupuncture for post-tonsillectomy pain: a randomized, double-blind, placebo-controlled trial. [77]	Randomized prospective trial	Intraoperative acupuncture is feasible, well tolerated, and results in improved pain and earlier return of diet after tonsillectomy.	Stanford Medical Scholars Program, Howard Hughes Medical Institute Medical Fellows Program, Stanford Children’s Health Research Institute Akiko Yamazaki and Jerry Yang Faculty Scholar
